# Orthogonal Printed Microstrip Antenna Arrays for 5G Millimeter-Wave Applications

**DOI:** 10.3390/mi13010053

**Published:** 2021-12-29

**Authors:** Muhammad M. Hossain, Md Jubaer Alam, Saeed I. Latif

**Affiliations:** 1 Department of Electrical and Computer Engineering, University of South Alabama, Mobile, AL 36688, USA; mh1934@jagmail.southalabama.edu; 2 Department of Systems Engineering, University of South Alabama, Mobile, AL 36688, USA; ma1925@jagmail.southalabama.edu

**Keywords:** MIMO, 5G, mmWave, scanning array, correlation coefficient

## Abstract

This article presents the design of a planar MIMO (Multiple Inputs Multiple Outputs) antenna comprised of two sets orthogonally placed 1 × 12 linear antenna arrays for 5G millimeter wave (mmWave) applications. The arrays are made of probe-fed microstrip patch antenna elements on a 90 × 160 mm^2^ Rogers RT/Duroid 5880 grounded dielectric substrate. The antenna demonstrates S_11_ = −10 dB impedance bandwidth in the following 5G frequency band: 24.25–27.50 GHz. The scattering parameters of the antenna were computed by electromagnetic simulation tools, Ansys HFSS and CST Microwave Studio, and were further verified by the measured results of a fabricated prototype. To achieve a gain of 12 dBi or better over a scanning range of +/−45° from broadside, the Dolph-Tschebyscheff excitation weighting and optimum spacing are used. Different antenna parameters, such as correlation coefficient, port isolation, and 2D and 3D radiation patterns, are investigated to determine the effectiveness of this antenna for MIMO operation, which will be very useful for mmWave cellphone applications in 5G bands.

## 1. Introduction

Smartphones and other electronic devices use specific frequencies in the radio frequency spectrum, typically under 6 GHz. These frequencies are starting to become crowded. Carriers can only squeeze so many bits of data on the same amount of radio frequency spectrum. As more devices come online, we are starting to see slower services and more dropped connections. The solution is to use the unused mmWave frequencies, such as 26 GHz (shorter millimeter wave falls between 10 to 300 GHz), that have never been used before for cellular communications [1,2,3,4,5,6,7,8,9,10,11,12,13,14,15,16]. In mmWave technologies, the antenna is an integral part for maintaining communications among mobile, fixed, and other handheld devices. However, the millimeter waves have higher propagation loss, and they tend to be absorbed by buildings and other obstacles. To achieve full functionalities necessary for true mmWave 5G communications, such as beam scanning, narrow beamwidth, high gain, etc., multiple phased arrays, or massive MIMO mmWave antennas are expected to be implemented in 5G cellular devices [17,18,19,20,21]. To achieve maximum coverage area and constructive interference in the direction of interest, the arrays need to be fed individually with different signals so that beams can be sent to different directions. Instead, the same signal with various amplitudes and different phases, commonly known as progressive phase shift, can be used between the radiating elements to steer beams in different angular directions to search for microcells to attain the strongest signal. Micro- and pico-cells will be implemented in 5G wireless systems to maximize connectivity and mitigate high propagation loss at high frequencies.

Another issue with mmWave communications is multipath and fading [22]. Each antenna is sensitive to only one type of polarization (horizontal or vertical polarization) at a time, and they cannot detect the actual polarization unless they are placed perpendicularly to each other. Arranging the arrays with orthogonal polarization assures polarization diversity [23,24]. It reduces coupling between the elements for better application in MIMO operations.

Several works have reported mmWave antenna designs with MIMO features for 5G applications. The work in [9] has proposed a low-profile 5G phased array antenna with unidirectional hemispherical beam coverage. The antenna size is 80.8 mm × 80.8 mm with a measured peak gain of 11.62 dBi. In [10], a four-layer metal stack-up structure is presented to achieve a peak gain of 17.37 dBi at 28 GHz where the target frequency range is 26.5–29.5 GHz. In [11], two types of mesh-grid phased-array antennas are presented featuring reconfigurable horizontal and vertical polarizations for smartphones. The design discussed in [12] has a 64-element dual-polarized phased-array antenna module for 28-GHz high-speed data communications. A two-port mmWave MIMO-based slot antenna with an electromagnetic bandgap (EBG) reflector has been proposed in [13], which has a peak gain of 11.5 dBi and improved MIMO performance. Another technique is presented in [14], where the bow-tie-shaped mmWave MIMO antenna is integrated with three pairs of metamaterial arrays to enhance the gain. A two-port mmWave MIMO antenna with an electromagnetic bandgap (EBG) is reported in [15]. The use of EBG with MIMO improves the antenna gain by 1.9 dBi with a peak gain of 6 dBi. In [16], a metamaterial structure is printed over a dielectric resonator antenna (DRA) with a four-element MIMO configuration. The introduction of metamaterial on top of DRA provides with about 13 dB better isolation compared to that without metamaterial. However, the maximum gain obtained is 7 dBi, which is relatively low.

It is important to have good bandwidth, better selectivity, and high gain among the signals to avoid interferences for 5G implementation [25,26,27]. Besides, it is evident from some of the studies that the overall gain with adequate scanning of the beam becomes half of the broadside gain when the arrays are scanned to 70° [28]. Most of the designs available in the literature are based on conventional planar array antennas where multiple sets of arrays are used to obtain MIMO. In our proposed work, a set of two orthogonal arrays are designed using the Dolph-Tschebyscheff excitation weighting presented in this article to achieve a larger gain with scanning beams and better polarization diversity performance, which will be eminently suitable for mmWave 5G cellular applications. Location of orthogonal arrays are chosen at two adjacent edges of a corner so that this antenna system can be used in the bezel area of a smartphone.

One of the goals of this research work is to design an antenna array for 5G wireless systems. The array is designed using microstrip patch antennas for the 26 GHz band with a return loss of at least −10 dB from 24–27 GHz. Beamforming of the array is accomplished using weighted excitation and phase shifting. A Dolph-Tschebysheff weighted excitation is used to achieve a reasonable gain with low sidelobe levels while scanning. In fact, this excitation method allows a low side-lobe level with narrower beamwidth. The array is scannable over the range of +/−45° from the broadside with a gain of greater than 12 dBi. The multiple input multiple output (MIMO) requirements of the 5G system will be satisfied because of the locations of the arrays in two different axes. The difference in polarization between the arrays will provide a low correlation coefficient, which is one of the requirements for a MIMO system. 

In this article, Section 1 explains the background study of the design as the introduction. Section 2 describes the construction of the antenna arrays as the antenna geometry. The detailed antenna array development is described in Section 3. Section 4 consists of results and discussion on different antenna parameters, such as scattering parameters, gain analysis, 3D radiation patterns, and correlation coefficient. Section 5 validates the performance of the antenna arrays in terms of scattering parameters and gains. Measured results from a fabricated prototype are also compared with the simulated ones. Section 6 has some concluding remarks.

## 2. Antenna Geometry

**Figure 1:**
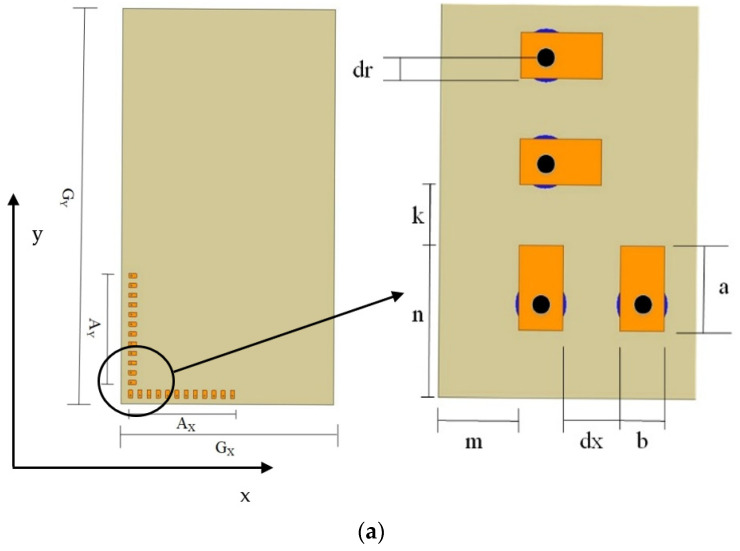

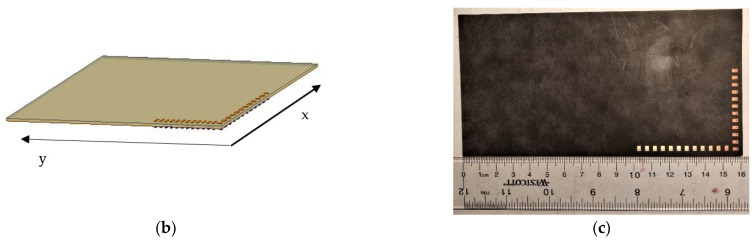
Geometry of the antenna arrays (**a**) top view, (**b**) 3D view, and (**c**) fabricated prototype.

Figure 1 depicts the geometry of the antenna. A set of two linear arrays, consisting of 12 microstrip patch antenna elements in each, are placed orthogonally over a dielectric substrate, Rogers RT/Duroid 5880, which has a dielectric constant of 2.2, loss tangent of 0.0009, and a thickness of 0.787 mm. The arrays are arranged at a corner of the grounded substrate (shown in Figure 1a). The entire substrate is 90 mm long (denoted by ‘*G_X_*’) and 160 mm wide (denoted by ‘*G_Y_*’), which is similar to the size of a modern-day smartphone. However, the area covered by the antenna arrays is only 56.5 × 6 mm^2^ along each side at one corner of the substrate, where each array length is 53.7 mm along the x and y-axes, denoted by ‘*A_X_*’ and ‘*A_Y_*’, respectively. The length and the width of each patch antenna are 3.1 mm and 2 mm, denoted by ‘*a*’ and ‘*b*’, respectively, as shown in Figure 1a (right). The feed probe is located 0.6 mm away from the center of each patch on the center line along its width. The edge-to-edge spacing between elements is 2.4 mm (denoted by ‘*k*’ and ‘*dx*’ in *y*-axis and *x*-axis, respectively), and the antennas are placed 2.5 mm (denoted by ‘*m*’) away from the edges. The distance from the edge of the ground plane to the inner edge of the patch at the corner is 5.9 mm (denoted by ‘*n*’), and from the edge of patch to its center is 1 mm (denoted by ‘*dr*’). The number of antenna elements and their orientation on the substrate are optimized to obtain a gain of 12 dBi or more for a large scanning range of +/−45°. Figure 1b,c shows the 3D view and the fabricated prototype of the structure, respectively. All the dimensions of the antenna arrays are listed in Table 1.

## 3. Development of the Array Antenna

In order to achieve the desired gain, linear arrays are designed and simulated using HFSS. Microstrip patch antenna elements are used in this regard. The microstrip patch antenna is simple and easy to implement in the restricted space of a mobile cellular unit. The aims in the antenna array design are to cover the frequency range from 24 GHz to 27 GHz with S_11_ values less than −6 dB and a gain of greater than 12 dBi over a scanning range of +/−45°. A Dolph-Tschebysheff excitation was chosen to keep sidelobe levels constant over the scanning range.

Using the single patch antenna element design, linear arrays are simulated using HFSS. For each array element, its excitation coefficient is calculated for a Dolph-Tschebyscheff distribution considering an even number of elements. The excitation coefficients for elements are found using the following array factor expression [29]:

(1) $$\;\;\;\;\;\;\;{\left(AF\right)}_{2M}=\overset{M}{\underset{n=1}{\sum }}a_{n}\cos\left[\left(2n-1\right)u\right]\;$$

where *a_n_* is the excitation coefficient of the *n*th element, and *M* = 2*N* with *N* being the number of elements. In this expression, *u* = *πd*cos *θ*/*λ*, where d is the element spacing.

A study is conducted for a linear array with various numbers of elements, and gain levels, half power beamwidth, and sidelobe levels (SLL) are compared. Each of these characteristics is presented in Table 2 for broadside radiation and for a scanning angle of 45°. Each array simulated has a uniform spacing between elements. A rigorous parametric study is done to attain the optimum distance between elements for maximum gain avoiding any grating lobes. The study shows that the best results can be obtained if the spacing between the elements is 0.39*λ* or 4.4 mm. To achieve at least 12 dBi gain and a scanning range of +/−45° with minimum sidelobe level, the 1 × 12 element array with 4.4 mm of element spacing is sufficient and is selected to keep the array size small. This spacing ensures maximum radiation towards a particular direction (or at a specific scanning angle) without any grating lobes. 

## 4. Results

In this section, the simulated results of the proposed antenna arrays are discussed. The structure is modeled, simulated, and studied in Ansys HFSS.

### 4.1. S-Parameter Analysis

**Figure 2:**
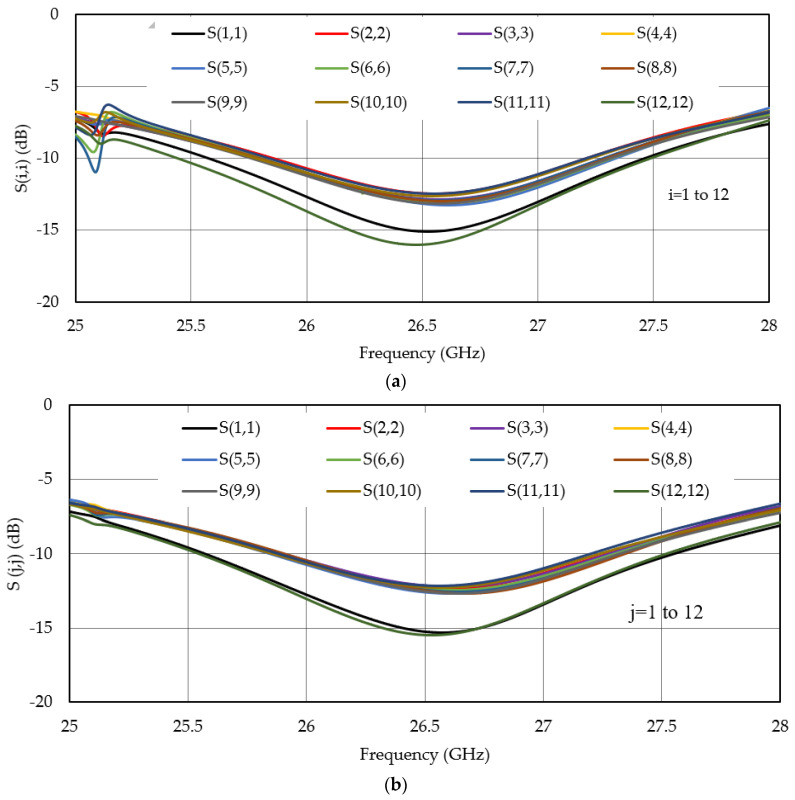
Scattering parameters: S(i,i) and S(j,j) of the antenna array elements for the (**a**) x-axis-oriented array and (**b**) y-axis-oriented array, respectively.

To quantify the antenna performance, scattering parameters or S-parameters are studied over the frequency range from 25 to 30 GHz. The simulation results show that the antenna has S_11_ below −10 dB from 25.5 GHz to 27.5 GHz with a minimum magnitude (reflection coefficient) of −17 dB at 26.5 GHz. S_11_ = −6 dB bandwidth is from 25 GHz to 28 GHz and beyond. The antenna arrays in both x-axis and y-axis are simulated, where the elements at the ends have better reflection coefficient with respect to other elements for both arrays. These results are obtained from Ansys HFSS. Figure 2a,b shows all the simulated results of reflection coefficients in the case of x-axis- and y-axis-oriented arrays, respectively.

### 4.2. Gain Analysis

One of the most important parameters of an antenna in 5G applications is its gain. High gain is desired in this application to overcome free-space path loss and greater selectivity of micro-base stations. To obtain the gain of the proposed antenna, the structure is simulated in both HFSS and CST, and the antenna arrays have shown a broadside gain of 13.3 dBi at the operating frequency 26.5 GHz. The main lobe of the antenna has a 3 dB beamwidth of 24°, whereas the first side lobe level is approximately −15 dB. The scanning of the antenna arrays is obtained by using a progressive phase shift, ϕ, between elements [29]:

(2) $$\phi =\left(kd+\frac{2.94}{N}\right)\sin\left(\theta \right)$$

θ=the desired scanning angle

**Figure 3:**
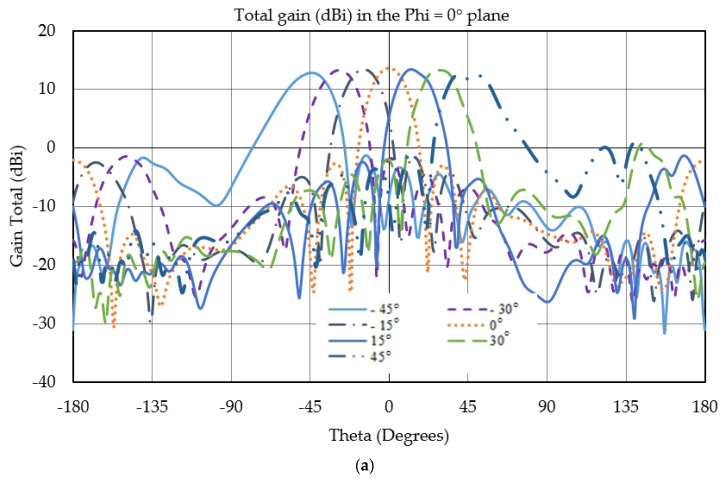

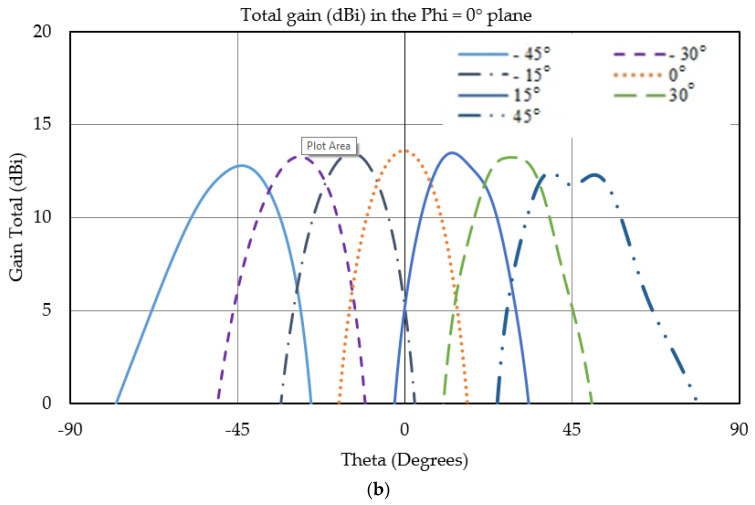
(**a**) Simulated array gains for various scanning angles in the elevation plane at 26.5 GHz. (**b**) Zoomed-in version of the gains for a smaller theta range.

The narrow beamwidth of the antenna mitigates high path loss and possesses better selectivity attenuating undesired signals. However, beamwidth increases as the main beam is scanned away from boresight. The arrays’ excitations are adjusted so that the main lobe can be scanned as far as possible maintaining a low sidelobe level and reasonable gain. Otherwise, it may increase the noise level. The maximum broadside gain is 14.1 dBi as can be noticed in Figure 3, whereas the minimum gain of the antenna is 12.9 dBi observed at a scanning angle of 45°. As the scanning angle increases, the gain drops and the beamwidth increase. Figure 3a exhibits the gain of the antenna arrays and Figure 3b shows zoomed-in inset of peak of total gain for various scanning angles; only the array along the x-axis is excited.

### 4.3. Radiation Pattern

**Figure 4:**
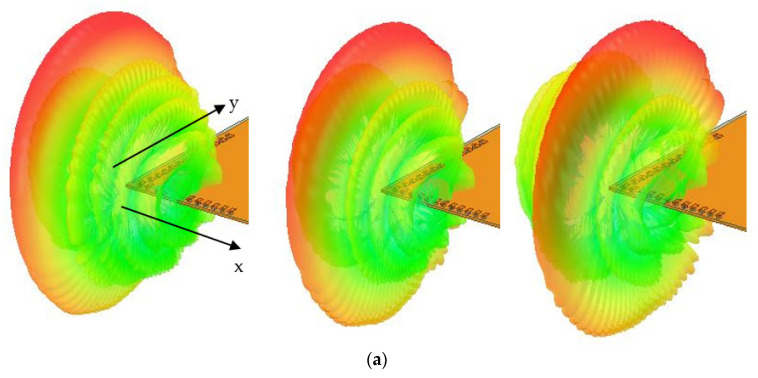

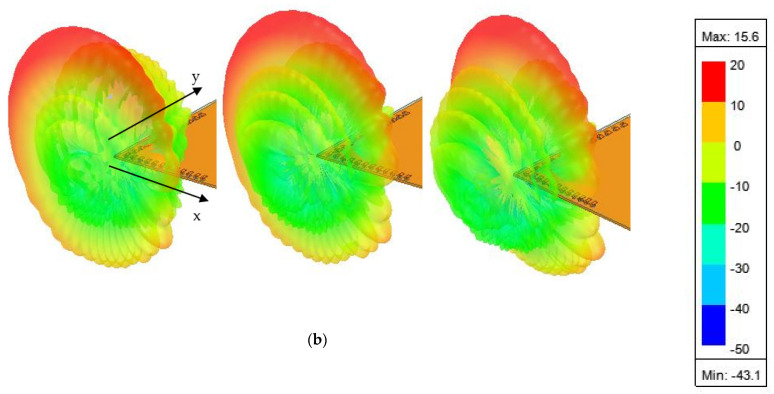
Simulated 3D radiation patterns for −30°, 0°, and 30° scanning angles: (**a**) when the x-axis-oriented array is excited and (**b**) when the y-axis-oriented array is excited.

Figure 4 presents the 3D radiation patterns of the antenna for −30°, 0°, and 30° scanning angles. When the array along the x-axis is excited, the scanning is achieved in the φ = 0° plane, and it is possible in the φ = 90° plane when the array along the y-axis is excited. While the beamwidth is narrow in the plane containing the array and main beam, it is wider in the other plan due to linear arrangement of the array elements. 

The parameter envelope correlation coefficient (ECC) determines the degree of isolation between the radiated fields of the arrays. The lower the ECC value, the more independently the two arrays will operate, which is beneficial for MIMO communication. However, the ECC cannot be calculated from the S-parameters of two independent arrays only. It requires a common field data, which can be formed by combining all the elements in the array. The ECC can be calculated by the following equation using the field data obtained from the simulation software [30]:

(3) $$\rho_{e}=\frac{{\left|\iint_{4\pi }^{\;}\left[\vec{F_{1}}\left(\theta ,\phi \right)*\vec{F_{2}}\left(\theta ,\phi \right)\right]d\Omega \right|}^{\;}}{\iint_{4\pi }^{\;}{\left|\vec{F_{1}}\left(\theta ,\phi \right)\right|}^{2}d\Omega \;\iint_{4\pi }^{\;}{\left|\vec{F_{2}}\left(\theta ,\phi \right)\right|}^{2}d\Omega \;}$$

**Figure 5:**
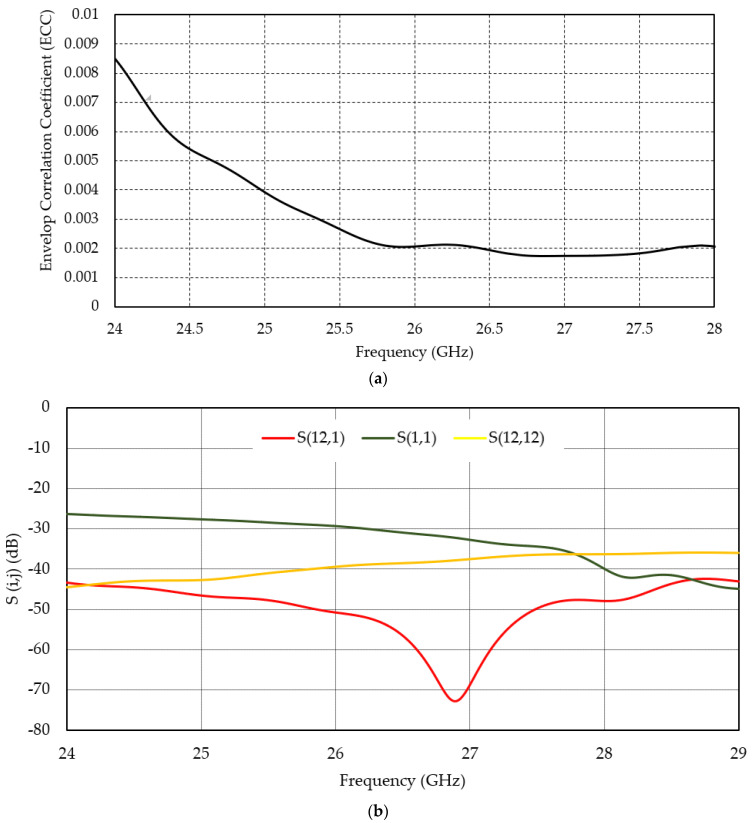
(**a**) Simulated correlation coefficient (ρ) between ports 1x and 1y (two closest ports), (**b**) S(i,j) performance of the orthogonal array.

Figure 5a shows the plot of ECC for two orthogonal arrays of the antenna. Since two sets of arrays are placed orthogonally to each other, the expected ECC value within the range would be approximately zero. The maximum value of the correlation coefficient can be found at the upper end of the band, which is 0.0065. In comparison to other designs, this ECC value less of than 0.0065 is quite low [30]. The orthogonal polarization of these two arrays ensures a good ECC performance from this arrangement. 

The transmission coefficient (S_ij_) values of the antenna support the ECC values (shown in Figure 5b). The lower negative values of S_ij_ confirm better isolation between two ports of the two array elements [31,32,33]. The isolation performance of the orthogonal arrays is evaluated between all two-port combination within the array and between elements in two orthogonal arrays, but some significant ones are presented in Figure 5b: S(i = 12, j = 1), S(i = 1, j = 1), and S(i = 12, j = 12). It is evident from the figure that the isolation between ports is very good, and as such, the interference will be very low. S_ij_ values are mostly below −30 dB in the operating frequency band for all combinations.

## 5. Validation

**Figure 6:**
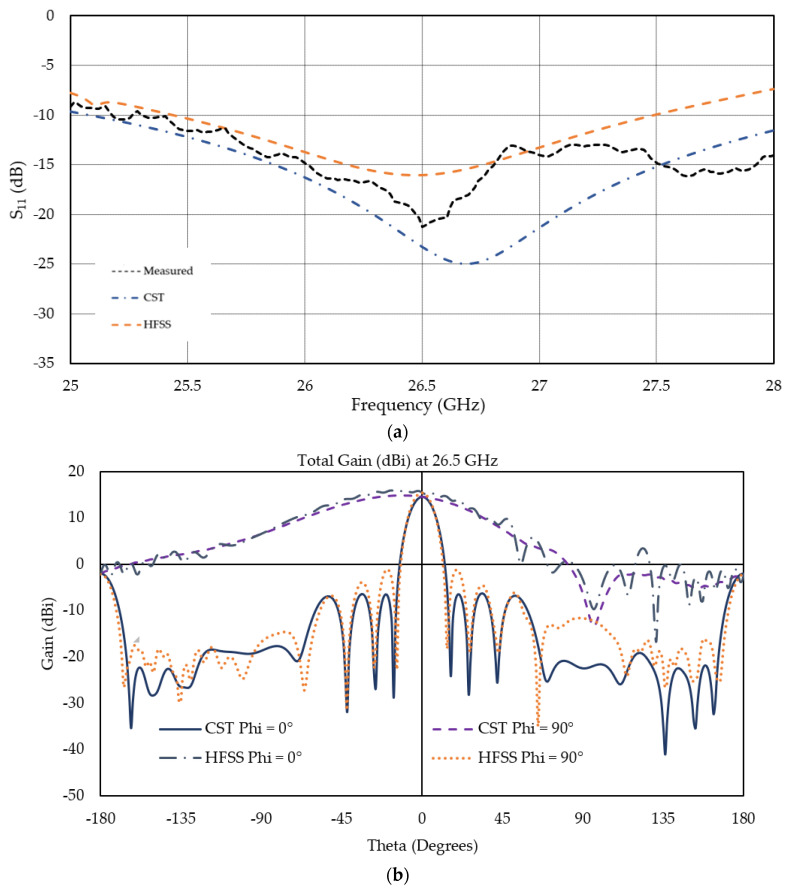
(**a**) S_11_ comparison among CST, HFSS, and measured values and (**b**) gain comparison obtained from CST and HFSS at 26.5 GHz.

The simulated scattering parameters obtained from Ansys HFSS are further verified by CST Microwave Studio. A fabricated prototype was developed in the Applied Electromagnetics Lab at the University of South Alabama using microfabrication technique, and reflection coefficients were measured using an Anritsu 37369A Vector Network Analyzer (VNA). The measured result of the antenna shows reasonable agreement with the simulated results, as shown in Figure 6a. Measured operating frequency bandwidth based on S_11_ =−10 dB criterion was over 2.4 GHz, from 25.6 GHz to 28 GHz or beyond. If S_11_ = −6 dB impedance bandwidth criterion is considered, S_11_ is below −6 dB for all three cases for a wide band of frequency and indicates a good agreement. The slight disagreement can be attributed to the uncertainty in the substrate permittivity at such a high frequency and fabrication errors.

Figure 6b exhibits the overall gain at the operating frequency of 26.5 GHz of the antenna array in the x-axis, which is calculated using both CST MWS and Ansys HFSS. The gains are plotted over the theta range from −180° to 180° in the φ = 0° and φ = 90° planes. Results obtained from both simulation tools are in very good agreement, confirming the accuracy of the results presented in this paper. 

Table 3 shows the comparison of various antenna parameters of the proposed work with some published designs. The main aim of the proposed design is to obtain MIMO performance with the use of two orthogonal linear arrays, which would take very little space on a mobile terminal. The orthogonal antenna arrays will fit in the bezel region at a corner of a smartphone. Most of the published works are based on conventional planar arrays. The overall size, gain, isolation, and ECC of the proposed antenna system are compared with those of some other antennas in Table 3. It can be observed that the proposed MIMO antenna array has good isolation and higher gain from a linear configuration compared to other designs presented in [1,13,14,15,16,34]. The ECC value is also quite good compared to that in other cases. 

## 6. Conclusions

The development and analysis of a 5G antenna consisting of two sets of orthogonally placed arrays are presented in this paper. The scattering parameters of the antenna arrays show good performance in the 5G band, confirmed by measured results. More than 12 dBi of gain over a scanning range of −45° to +45° in two planes allows the antenna to work effectively in mmWave 5G systems with less multipath fading and good selectivity opportunity for available base stations. Good ECC (below 0.0065) values for arrays and excellent isolation performance indicate that the arrays arranged in the antenna system will operate well in MIMO communications. The formation of the antenna arrays creates a high degree of isolation by orthogonal polarization between them, which makes the antenna more desirable in mmWave applications.

## Figures and Tables

**Table 1:** Parameters of the antenna arrays (dimensions in mm).

*G_X_*	*G_Y_*	*A_X_*	*A_Y_*	*k*	*m*	*n*	*a*	*b*	*dx*	*dr*
90	160	53.7	53.7	2.4	2.5	5.9	3.1	2	2.4	1

**Table 2:** Linear array characteristics by number of elements.

Array Size	Broadside			45° Scan		
*X* × *Y*	Gain (dB)	HPBW (°)	SLL (dB)	Gain (dB)	HPBW (°)	SLL (dB)
1 × 4	9.93	34.76	−15.33	NC	NC	NC
1 × 6	11.02	22.19	−13.48	9.85	30.84	−7.07
1 × 8	12.06	17.35	−13.66	11.28	26.59	−11.37
1 × 10	13.1	13.29	−13.86	12.26	22.15	−11.8
1 × 12	14.1	12.25	−16.16	12.9	21.43	−16.1
1 × 14	14.3	9.79	−15.33	13.45	18.04	−13.73

NC: Not Calculated.

**Table 3:** Comparison with other published works.

Ref.	Substrate Material	Area (mm^2^)	Optimization Techniques	Operating Freq. (GHz)	Gain (dBi)	Isolation (dB)	ECC
[13]	Rogers RO4003	20 × 53	MIMO	28, 38	11.5, 10.9	20	<0.12
[14]	Rogers RT/duroid 5880	30 × 30.5	MIMO + EBG	26	7.4	N/A	N/A
[15]	Rogers RT/duroid 6002	20 × 60	DRA-based MIMO	24	6	37	0.24
[16]	Rogers RT/duroid 5880	20 × 40	MIMO	28	7	29.34	0.02
[1]	Rogers RT/duroid 5880	21 × 85	MIMO	26	10.27	45	0.004
[34]	100 μm glass	28.9 × 16.7	Minimal matching	27	9.51	N/A	N/A
Proposed work	Rogers RT/duroid 5880	X-Y arrays 53.5 × 3.1 3.1 × 53.5	MIMO	26.5	14	45	<0.002
